# The Impact and Mechanism of Methylated Metabotropic Glutamate Receptors 1 and 5 in the Hippocampus on Depression-Like Behavior in Prenatal Stress Offspring Rats

**DOI:** 10.3390/jcm7060117

**Published:** 2018-05-23

**Authors:** Tianwei Lin, Shaokang Dang, Qian Su, Huiping Zhang, Junli Zhang, Lin Zhang, Xiaoxiao Zhang, Yong Lu, Hui Li, Zhongliang Zhu

**Affiliations:** 1 Shaanxi Province Biomedicine Key Laboratory, College of Life Sciences, Northwest University, Xi'an 710069, Shaanxi, China; lintianwei@stumail.nwu.edu.cn (T.L.); zhangjunli@stumail.nwu.edu.cn (J.Z.); z-lin@stumail.nwu.edu.cn (L.Z.); zhangxiaoxiao@stumail.nwu.edu.cn (X.Z.); 2 Department of Pharmacology, College of Medicine, Xi’an Jiaotong University, Xi’an 710061, Shaanxi, China; dskang121316@stu.xjtu.edu.cn (S.D.); suqian@mail.xjtu.edu.cn (Q.S.); zhanghuiping@mail.xjtu.edu.cn (H.Z.); luyong@mail.xjtu.edu.cn (Y.L.); huili@mail.xjtu.edu.cn (H.L.)

**Keywords:** prenatal stress (PS), methylation, mGluR1, mGluR5, depression

## Abstract

An increasing number of epidemiological investigations and animal models research suggest that prenatal stress (PS) could cause depression-like behavior in the offspring, which is sex specific. However, the underlying mechanisms remain to be elucidated. This study is to investigate the promoter methylation of metabotropic glutamate receptor 1 (mGluR1) and metabotropic Glutamate Receptor 5 (mGluR5) gene modification on PS induced depression-like behavior in offspring rats (OR). PS models were established, with or without 5-aza-2′-deoxycytidine (5-azaD, decitabine) treatment. Animal behavior was assessed by the sucrose preference test (SPT), forced swimming test (FST), and open field test (OFT). The mRNA and protein expression levels of mGluR1 and mGluR5 in the hippocampus of offspring were detected with quantitative real-time PCR and Western blot analysis, respectively. The promoter methylation in the hippocampus of mGluR1 and mGluR5 OR were also analyzed. SPT showed significantly reduced sucrose preference in PS induced OR. FST showed significantly prolonged immobility time in PS induced OR. OFT showed significantly reduced central residence time in PS induced OR and no significantly influence in rearing as well as in frequency of micturition. Moreover, the mRNA, protein expression levels, and gene promoter methylation level of mGluR1 and mGluR5 in the hippocampus were significantly increased in the PS induced male OR, while no significantly influence in the PS induced female OR. Furthermore, the PS induced effects in male OR could be reversed by the microinjection of 5-azaD. In conclusion, our results showed that the promoter methylation of mGluR1 and mGluR5 gene modification is only involved in PS induced depression-like behavior in male OR in a sex-specific manner. These findings might contribute to the understanding of the disease pathogenesis and clinical treatment in future.

## 1. Introduction

The main clinical manifestations of depression, also known as depression obstacles, are symptoms related to low sentiment and being low-spirited along with decreased interests for external activities. These behavioral symptoms may also cause suicidal behavior or attempts [1]. There are at least 350 million people in the world tormented by varying degrees of depression [2]. According to the World Health Organization, depression will become the second leading cause of disability around the world by the year 2020 [3].

Mammalian brains are very susceptible to stress during the process of fetal development. The latest research reflects that prenatal stress is certainly related to the development of adverse nerves in the brains of their offspring. This includes affective disorders, such as attention deficit, autism, schizophrenia, and anxiety as well as depression [4,5,6]. The disorder of the glutamate system in the central nervous system may be involved in such pathological effects of stress or stress-related psychiatric disorders [7].

Previous experimental studies show that glutamate and its receptor play a crucial role in the pathogenesis of stress-related psychiatric disorders [8]. For example, glutamate and its receptors play a key role in the production of stress related diseases, such as depression and anxiety disorders [9]. Studies have shown that mGluRl and mGluR5 are related to a variety of mental disorders, such as Parkinson’s syndrome, Alzheimer’s disease, anxiety, and depression [10,11,12]. Prenatal stress can impair the ionotropic glutamate receptors dependent synaptic plasticity of neurons, which is reliant on metabotropic glutamate receptors, leading to a decline in both learning and memory functions [13,14,15]. Abnormal expression of glutamate in the brain and plasma of depression patients has been confirmed by a number of studies. For example, an autopsy report showed that the volume of glutamate in the prefrontal lobe of patients with bipolar affective disorder and severe depression significantly increased [16].

As a means to avoid the influence of females in estrus caused by hormonal changes, previous studies conducted regarding the effects of prenatal stress-induced behavior on anxiety and depression were concentrated in the male offspring. Throughout numerous animal experiments [5,17], it was reported that there was no difference in male offspring immobile time with regard to the effects of prenatal stress after the display of gender differences in both male and female offspring. However, the time of immobility of the female offspring increased during the forced swimming test [18]. In contrast, some other studies have shown that in the forced swimming test, after prenatal stress, only the time of immobilization of male offspring was increased when compared to the control group [19,20]. Recent studies suggest that prenatal stress may influence the expression of glutamate receptor protein within the hippocampus, prefrontal cortex, and the amygdala of both male and female offspring. However, there have been few studies conducted as a means to compare the differences in the expression of glutamate receptor protein in female and male offspring. Prenatal stress resulted in the decrease of NR1 protein levels [21], mGluR5 mRNA expression, and the increase in NR2B protein levels in the hippocampus of the male offspring [22], while there was no effect on the female offspring. Zuena’s laboratory studies have shown that prenatal stress can lead to a decrease in the expression level of mGluR5 in the hippocampus of the male offspring [23].

DNA methylation is one of the earliest discovered DNA modification methods and is also a well-studied epigenetic modification method. Within mammals, methylation occurs only at cytosine—that is the 5′ end of the cytosine dinucleotide CpG converted into 5′ which is methylcytosine under the DNA methyltransferases action (DNMTs) [24]. Generally, the CpG locus is not particularly common in the genome, and is also closely related to the location of the gene promoter, known as the CpG Island [25]. Methylation of the CpG site can have significant effects on gene expression. Especially numerous studies have shown that DNA methylation is involved in various types of cellular processes. For instance, it can induce chromatin conformation, DNA conformation, DNA stability, and the interaction between DNA and protein, thereby controlling gene expression [26]. Moreover, DNA methylation usually inhibits gene expression, while demethylation induces the reactivation and expression of the gene. This method of DNA modification is performed as a means to achieve the regulation of gene expression without changing the gene sequence [27,28]. DNA methylation can be directly inherited or cross-generationally inherited to the offspring [29]. Within depressed patients, the levels of DNA methylation in the GLUT1 and GLUT4 core promoter regions were considerably altered [30].

DNA methylation has been demonstrated to silence the expression of many genes and the event is reversible by specific inhibitors of DNA methylation, such as 5-aza-2′-deoxycytidine (5-azaD, decitabine) [31,32,33]. Studies have shown that 5-azaD can improve depressive behavior, accompanied by a decrease in DNA methylation and increase of BDNF expression. Moreover, in the forced swimming (FST) experiment, microinjection of 5-azaD into the hippocampus of rats can produce antidepressant effect [34].

In this study, whether the metabotropic glutamate receptors 1 and 5 (mGluR1 and mGluR5) promoter region methylated modification involved in the PS induced depression-like behavior in the offspring rats (OR), and the related mechanisms, were explored. The PS induced depression-like behavior was detected by the sucrose preference test (SPT), forced swimming test (FST), and open field test (OFT). The mRNA and protein expression levels of mGluR1 and mGluR5 in the OR hippocampus were evaluated. The promoter methylation of mGluR1 and mGluR5 Gene levels in the OR hippocampus were also detected. The effects of JNJ16259685 (JNJ), a selective mGluR1 antagonist, and MTEP, a metabotropic glutamate receptor subtype 5 Antagonists on the PS induced depression-like behavior in the OR were investigated.

## 2. Materials and Methods

### 2.1. Animals

Virgin Sprague-Dawley (SD) rats were provided by the Experimental Animal Centre of Xi’an Jiao Tong University School. These animals were housed with free access to food and water at a constant temperature of 20 ± 2 °C, under a 12 h light/dark cycle (lights were on at 8:00 a.m.). Female rats (weighing 230–250 g) were placed overnight with adult male rats (weighing 280–300 g) for mating (rate of 3:1). The vaginal smear was examined on the following morning. The day on which the smear was positive for the sperm detection was determined as the embryonic day 0. Then the pregnant rats were separately housed. All animals were treated in accordance with the guidelines of the Centre for the Humane Treatment of Animals. Moreover, attempts were also made as a means to minimize the number of animals used.

### 2.2. Animal Model Establishment

**Figure 1:**
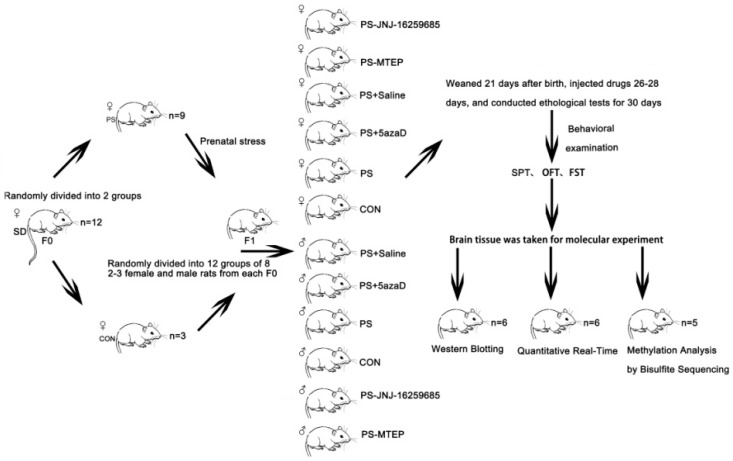
Pregnant rats (*n* = 12) randomly divided into two groups the CON (*n* = 3) and the PS group (*n* = 9). After the offspring were weaned on postnatal day the OR randomly divided into 12 groups (*n* = 8), female and male respectively. At 30 days after birth, depression-like behavior was assessed by the SPT, FST, and OFT (*n* = 8 each group). Then, the mRNA and protein expression levels (*n* = 6 each group) and the promoter methylation (*n* = 5 each group) of mGluR1 and mGluR5 in the OR hippocampus were evaluated.

Prenatal stress (PS) in rats is a rather good model of early stress and can induce long-lasting neurobiological and behavioural alterations [35]. The pregnant rats (*n* = 12) were randomly divided into two groups: the control group (CON, *n* = 3) was left undisturbed throughout the pregnancy; and the prenatal stress group (PS, *n* = 9), which was exposed to restraint stress, three times a day from day 14 to day 20, 45 min each time [36,37]. To avoid these animals from being habituated to the daily program, phases were randomly shifted within certain time periods and the interval between the durations was at least 2 h. The prenatal stress groups were placed into a small plastic cage (Ø6 × 17–18 cm). The cage at both ends of each vent was fixed at the bottom, and the other end was adjusted based on the length of the rat’s body. After birth, for all the groups, offspring were housed with their natural mothers. Litters containing 10–14 pups with comparable numbers of rats were used for the following experiments. After the offspring were weaned on postnatal day (PND) 21, male and female pups were separately housed. Two or three male OR and female OR were randomly selected from each litter and divided into six groups (CON, PS, PS-5azaD, PS-Saline, PS-JNJ-16259685 (a selective metabotropic glutamate receptor 1 antagonist), and PS-MTEP (a metabotropic glutamate receptor subtype 5 antagonists), *n* = 8 each group) and tested for either the behavioral examination or the corresponding index test after 9 days. On postnatal day (PND) 26, 5azaD, Saline, JNJ-16259685, MTEP was injected to the OR hippocampus, respectively. After 3 days, depression-like behavior was assessed by the sucrose preference test (SPT), forced swimming test (FST), and open field test (OFT) (*n* = 8 each group). The rats were only administered once to the FST and OFT. Then, the mRNA and protein expression levels of mGluR1 and mGluR5 in the OR hippocampus were evaluated (*n* = 6 each group), and the promoter methylation of mGluR1 and mGluR5 gene level in the OR hippocampus was detected (*n* = 5 each group). The Figure 1 diagram describes the experimental steps.

### 2.3. Drug Treatment

At 3 days before the depression-like behavior experiment, offspring were subjected to the stereotaxic surgery as previously described [38]. Offspring received microinjections of 2 μL 5azaD (90 ng/μL), Saline, JNJ (90 ng/μL), or MTEP (90 ng/μL) to the bilateral hippocampus (AP: −1.3 ± 0.1 R: 2.0 ± 0.2 H: 3.5 ± 0.2) mm, according to the Paxinos and Watson coordinates [39]) for 3 days, for the PS-5azaD, PS-Saline, PS-JNJ-16259685, and PS-MTEP groups, respectively. Injections were given to the rats with a microdriver for approximately 10–15 min. After injection, the rats were all treated with antibiotic (gentamicin) as a means to prevent infection until after waking up to a cage feeding.

### 2.4. Sucrose Preference Test, SPT

All rats were given 200 mL 2% sucrose solution (*w*/*v*) along with drinking water at the same time. Drinking and drinking water was prohibited for three hours before the experiment. Total consumption was determined by weighing the sucrose solution bottle before and after each 2 h session. In order to prevent rat inertia, the sucrose solution and water bottle should be alternated in position every 1 h. The experiment was performed three times according to this method. With the sucrose preference degree being a measure of depression behavior, the difference compared to the experimental group was then analyzed. Sucrose preference (percent) was calculated as follows: sucrose solution consumption/(water consumption + sucrose solution consumption) × 100%.

### 2.5. Open Field Test, OFT

An area measured at 40 × 40 × 35 cm was used for the open field test (25 same squares (8 × 8 cm) at the bottom of the box). The rats were put in the middle of the open box. All the movements in the 6 min free inquisitive space were recorded by a video camera. The center time (at least 3 paws enter the central region), rearing (two front paws on the walls or with only two claws standing), and frequency of micturition was manually measured by a trained analyzer. After one experiment, the rats were put back and the whole open box was wiped with 5% ethanol to remove the residual odor of the experimental rats and avoid the effect on the next open field experiment.

### 2.6. Forced Swimming Test, FST

FST was performed according to previous reports [40]. The experimental rats were placed in a 50 cm high, 20 cm inner diameter of the cylindrical tank. The tank contained 40 cm of water 25 ± 1 °C. Throughout the course of the experiment, a quiet and calm atmosphere was maintained. The experiment observed the rats swimming within a 10 min period and the immobility time (the sum of time without any action and floating on the water surface) was recorded.

### 2.7. Separation of the Hippocampus

After the behavioral test, the rats were narcotized with a 10% chloral hydrate standard solution (1.2–1.5 mL/100 g body weight) and decapitated. The hippocampal tissues were rapidly split on ice and were subsequently stored in liquid nitrogen, while the detached tissues were kept in a −80 °C refrigerator until further analysis.

### 2.8. Quantitative Real-Time RT-PCR

Total RNA of the hippocampal tissues were extracted by the RNA fast 200 kit (Fastagen, Shanghai, China). Then, reverse transcription was conducted where 1000 nanograms of RNA from each sample were reverse transcribed. Moreover, the reverse transcription reaction was performed utilizing the Revert Aid First Strand cDNA Synthesis Kit (Thermo, Waltham, MA, USA). The reaction was performed briefly in the GeneAmp PCR System 9700 (Bio-Rad, Hercules, CA, USA). The quantitative real-time PCR was performed in the CFX Connect Real-Time PCR (Bio-Rad, Hercules, CA, USA). Quantitative real-time PCR assays were conducted using Thunderbird TM SYBR^®^ qPCR Mix (Toyobo, Osaka, Japan). The specific gene primers were then synthesized by the Sangon Biological Engineering Co., Ltd. (Shanghai, China). Moreover, the production lengths of primer sequences utilized in this experiment are reflected in Table 1. The relative expressions of mGluR1 and mGluR5 were thus calculated through the adaptation of 2^−∆ΔCt^ methods.

### 2.9. Western Blotting

Total protein of the hippocampal tissues were extracted by the Total Extraction Sample Kit (Sigma, Shanghai, China) and the protein concentration was determined by the BCA assay (Bio-Rad Hercules, CA, USA). Two hundred micrograms of each sample protein were separated through SDS–PAGE electrophoresis utilizing a 10% running gel and then transferred to the 0.45 um polyvinylidene difluoride (PVDF) membrane (Millipore, Waltham, MA, USA). The membranes were then incubated for 2 h at room temperature in 5% fat-free dry milk in phosphate buffered saline with a 0.1% Tween-20 (PBST). Immunoblotting was carried out overnight at a temperature of 4 °C with the specific antibody titer in the serum of rabbits immunized with the purified protein mGluR1 was 1:3000 (Abcam, Cambridge, UK) and mGluR5 was 1:1000 (Abcam, Cambridge, UK) in PBST buffer. The membranes were stripped three times with PBS-Tween-20 0.1% and incubated with the appropriate HRP-conjugated goat antirabbit secondary antibody (1:10,000, Pioneer, Xi'an, Shaanxi, China) at room temperature for 2 h. After three rinsing cycles in the PBST buffer, protein bands were visualized with an enhanced chemiluminescence kit (Pierce, Holmdel, NJ, USA), and the lanes were calculated by the Image Lab software (Bio-Rad, Hercules, CA, USA).

### 2.10. Methylation Analysis by Bisulfite Sequencing PCR

Total DNA of hippocampal tissues was extracted, respectively, by DNA extraction kit (Sangon Biotech, Shanghai, China). The genomic DNA were modified by bisulfite sodium by BisulFlash DNA Modification Kit (Epigentek, Farmingdale, NY, USA), and PCR amplification. PCR electrophoresis and recovery was by SanPrep Column Plasmid Mini-Preps Kit (Sangon Biotech, Shanghai, China). PCR-purified products are connected to pUC18-T carrier. Competent cells were prepared by Rapid Competent Cell Preps Kit (Sangon Biotech, Shanghai, China). Each transformed clone was placed onto LB plates which contained ampicillin/IPTG/X-gal exactly as described in the manufacturer´s instructions and subjected to overnight growth at 37 °C. Plasmid DNA extraction was by SanPrep Column Endotoxin-Free Plasmid Mini-Preps Kit (Sangon Biotech, Shanghai, China). Sequencing was completed by Sangon Biological Engineering Co., Ltd. (Shanghai, China). The specific gene primers were synthesized by the Sangon Biological Engineering Co., Ltd. (Shanghai, China). Moreover, the production lengths of the primer sequences used in this experiment are shown in Table 2.

### 2.11. Statistics

All values reported were mean ± SEM. Data analysis was performed using the SPSS 20.0 software. For data with variance homogeneity and normal distribution, the two-way ANOVA was performed. Additionally, one-way ANOVA were used to analyze the sex differences. *p* < 0.05 was considered statistically significant.

## 3. Result

### 3.1. 5-azaD Treatment Group Showed Significant Antidepressant Effect

**Figure 2:**
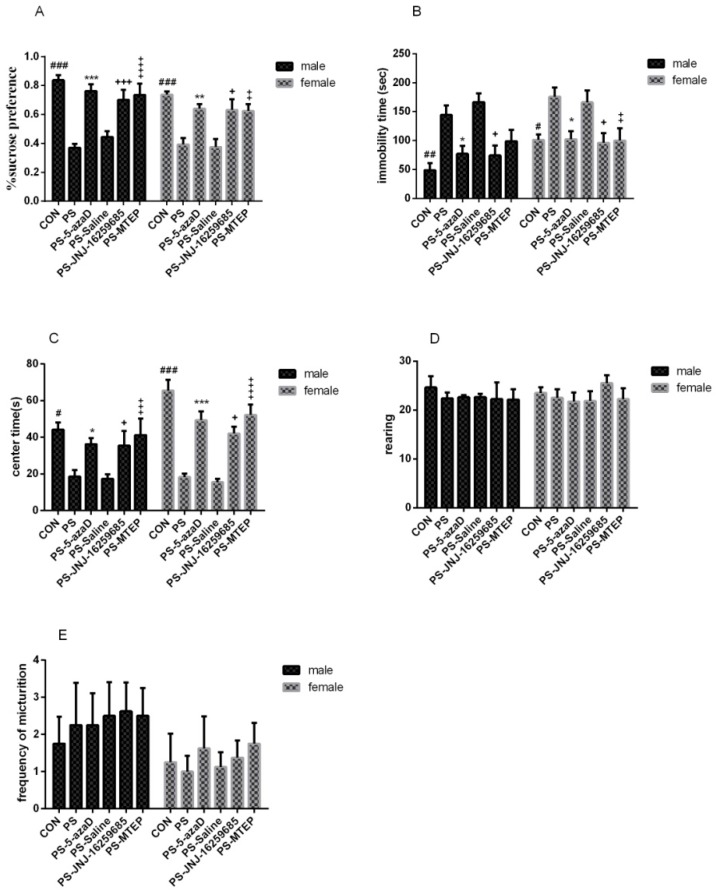
The depression-like behavior test of OR. (**A**) Sucrose preference in PS induced OR; (**B**) Immobility time in PS induced OR; (**C**–**E**) Center time, rearing, and frequency of micturition shown in six groups (*n* = 8 each group). Data is expressed as mean ± SEM. ^###^ *p* < 0.001, ^##^ *p* < 0.01, ^#^ *p* < 0.05 compare with CON and *** *p* < 0.001, ** *p* < 0.01, * *p* < 0.05 compare with PS. ^+++^ *p* < 0.001, ^+^ *p* < 0.05 compare with PS. 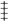 *p* < 0.001, 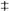 *p* < 0. 05 compare with PS.

Previous studies have reported that prenatal stress significantly reduced the sugar preference (2%) of the OR (Sun H., et al., 2013). Therefore, in this study, the sucrose preference test was used to test the ability of the rats to respond to rewards. For experiment 1 (Figure 2A), based on the one-way ANOVA, sex differences were observed (F_(1,94)_ = 3.148, *p* = 0.034 < 0.05), as well as the overall effects of male (F_(5,42)_ = 12.750, *p* < 0.001) and female (F_(5,42)_ = 9.328, *p* = 0.013 < 0.05). Our results in the male group analysis showed that, when compared with the CON group, the sugar solution consumption in the PS group was remarkably decreased (*p* < 0.001), with no difference in the PS-5azaD group (*p* = 0.913 > 0.05). Then, compared with the PS group, the sugar solution consumption in the PS-5azaD group (*p* < 0.001), PS-JNJ group (*p* = 0.008 < 0.01), and PS-MTEP group (*p* < 0.001) was significantly increased, with no difference in the PS-Saline group (*p* = 0.915 > 0.05). The female group analysis showed that, when compared with the CON group, the sugar solution consumption in the PS group was remarkably decreased (*p* < 0.001), with no difference in the PS-5azaD group (*p* = 0.716 > 0.05). Then, compared with the PS group, the sugar solution consumption in the PS-5azaD group (*p* = 0.09 < 0.01), PS-JNJ group (*p* = 0.013 < 0.05), and PS-MTEP group (*p* = 0.018 < 0.05) was significantly increased, with no difference in the PS-Saline group (*p* = 0.948 > 0.05).

The effects of PS on the immobility time of OR and the effect of 5-azaD were then analyzed. For experiment 1 (Figure 2B), based on the one-way ANOVA, sex differences were observed (F_(1,94)_ = 0.061, *p* = 0.805 > 0.05), as well as the overall effects of male (F_(5,42)_ = 7.535, *p* < 0.001) and female (F_(5,42)_ = 3.808, *p* = 0.006 < 0.01). Our results in the male group analysis showed that, when compared with the CON group, the nonmovement time in the PS group was remarkably decreased (*p* = 0.002 < 0.01), with no difference in the PS-5azaD group (*p* = 0.713 > 0.05). Then, compared with the PS group, the nonmovement time in the PS-5azaD group (*p* = 0.046 < 0.05) and PS-JNJ group (*p* = 0.034 < 0.05) was significantly increased, with no difference in the PS-Saline group (*p* = 0.917 > 0.05) and PS-MTEP group (*p* = 0.341 > 0.05). The female group analysis showed that, when compared with the CON group, the nonmovement time in the PS group was remarkably decreased (*p* = 0.034 < 0.05) with no difference in the PS-5azaD group (*p* = 1.00 > 0.05). Then, compared with the PS group, the nonmovement time in the PS-5azaD group (*p* = 0.038 < 0.05), PS-JNJ group (*p* = 0.02 < 0.05), and PS-MTEP group (*p* = 0.03 < 0.05) was significantly increased, with no difference in the PS-Saline group (*p* = 0.998 > 0.05).

The effects of PS on the center time of OR and the effect of 5-azaD were then analyzed. For experiment 1 (Figure 2C), based on the one-way ANOVA, sex differences were observed (F_(1,94)_ = 4.086, *p* = 0.046 < 0.05), as well as the overall effects of male (F_(5,42)_ = 3.680, *p* = 0.008 < 0.01) and female (F_(5,42)_ = 19.360, *p* < 0.001). Our results in the male group analysis showed that, when compared with the CON group, the center time in the PS group was remarkably decreased (*p* = 0.02 < 0.05), with no difference in the PS-5azaD group (*p* = 0.317 > 0.05). Then, compared with the PS group, the center time in the PS-5azaD group (*p* = 0.031 < 0.05), PS-JNJ group (*p* = 0.038 < 0.05) and PS-MTEP group (*p* = 0.006 < 0.01) was significantly increased, with no difference in the PS-Saline group (*p* = 0.875 > 0.05). The female group analysis showed that, when compared with the CON group, the center time in the PS group was remarkably decreased (*p* < 0.001), with no difference in the PS-5azaD group (*p* = 0.123 > 0.05). Then, compared with the PS group, the center time in the PS-5azaD group (*p* < 0.001), PS-JNJ group (*p* = 0.013 < 0.05), and PS-MTEP group (*p* < 0.001) was significantly increased, with no difference in the PS-Saline group (*p* = 0.972 > 0.05).

The effects of PS on the rearing of OR and the effect of 5-azaD were then analyzed. For experiment 1(Figure 2D), based on the one-way ANOVA, sex differences were observed (F_(1,94)_ = 0.014, *p* = 0.906 > 0.05). In addition, two-way ANOVA showed that there was no significant difference in the rearing between each male (F_(5,42)_ = 0.220, *p* = 0.952 > 0.05) and female (F_(5,42)_ = 0.632, *p* = 0.677 > 0.05) group.

The effects of PS on the frequency of micturition of OR and the effect of 5-azaD were then anaylzed. For experiment 1 (Figure 2E), based on the one-way ANOVA, sex differences were observed (F_(1,94)_ = 5.350, *p* = 0.023 < 0.05). In addition, two-way ANOVA showed that there was no significant difference in the frequency of micturition between each male (F_(5,42)_ = 0.129, *p* = 0.985 > 0.05) and female (F_(5,42)_ = 0.228, *p* = 0.948 > 0.05) group.

### 3.2. mGluR1 and mGluR5 mRNA Expression in the Hippocampus

**Figure 3:**
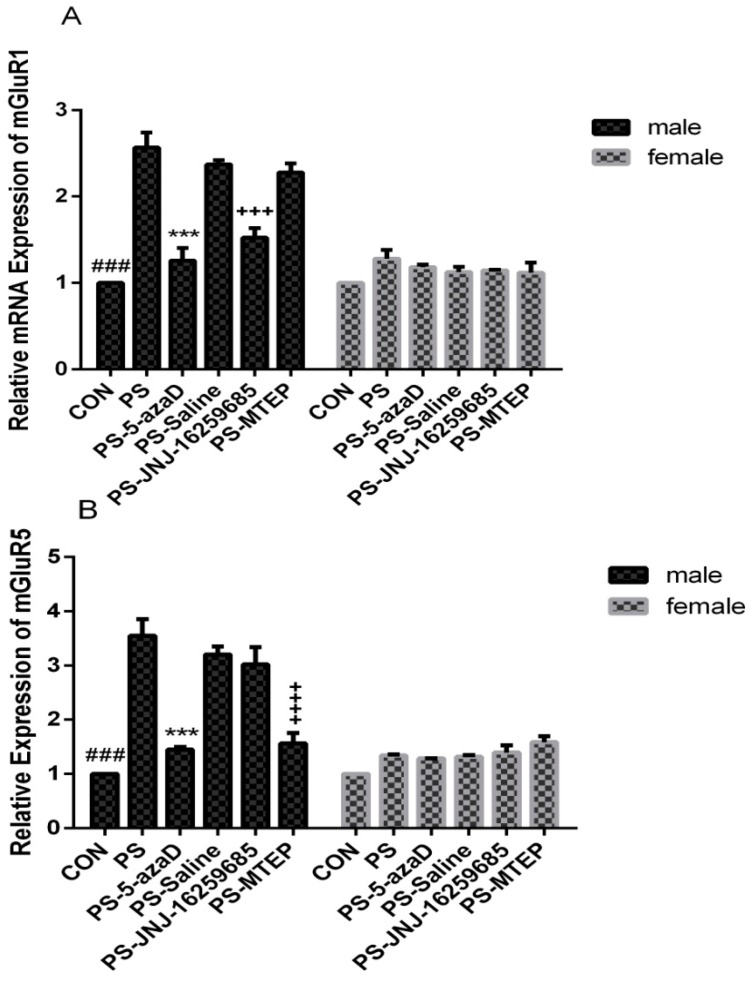
QRT-PCR analysis of mGluRl and mGluR5 mRNA levels in the hippocampus shown in six groups (*n* = 6 each group). Data is expressed as mean ± SEM. ^###^ *p* < 0.001 compare with CON. *** *p* < 0.001, ^+++^ *p* < 0.001, 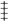 *p* < 0.001 compare with PS.

The effects of PS on the mRNA and protein expression levels of mGluR1 and mGluR5 were studied by quantitative real-time PCR and Western blot analysis, respectively. For experiment 2 (Figure 3A), based on the one-way ANOVA, sex differences were observed (F_(1,70)_ = 36.814, *p* < 0.001), as well as the overall effects of male (F_(5,30)_ = 33.117, *p* = 0.011 < 0.05). Our results in the male group analysis showed that, when compared with the CON group, the mGluR1 mRNA expression in the PS group was remarkably increased (*p* < 0.001), with no difference in the PS-5azaD group (*p* = 0.608 > 0.05). Then, compared with the PS group, the mGluR1 mRNA expression in the PS-5azaD group (*p* < 0.001) and PS-JNJ group (*p* < 0.001) was significantly decreased, with no difference in the PS-Saline group (*p* = 0.829 > 0.05) and PS-MTEP group (*p* = 0.485 > 0.05). In addition, two-way ANOVA showed that there was no significant difference in mGluR1 mRNA expression between each female (F_(5,30)_ = 1.871, *p* = 0.129 > 0.05) group.

The effects of PS on the mGluR5 mRNA expression were investigated. For experiment 2 (Figure 3B), based on the one-way ANOVA, sex differences were observed (F_(1,70)_ = 26.969, *p* < 0.001), as well as the overall effects of male (F_(5,30)_ = 27.702, *p* < 0.001). Our results in the male group analysis showed that, when compared with the CON group, the mGluR5 mRNA expression in the PS group was remarkably increased (*p* < 0.001), with no difference in the PS-5azaD group (*p* = 0.636 > 0.05). Then, compared with the PS group, the mGluR5 mRNA expression in the PS-5azaD group (*p* < 0.001) and PS-MTEP group (*p* < 0.001) was significantly decreased, with no difference in the PS-Saline group (*p* = 0.836 > 0.05) and PS-JNJ group (*p* = 0.465 > 0.05). In addition, two-way ANOVA showed that there was no significant difference in mGluR5 mRNA expression between each female (F_(5,30)_ = 2.268, *p* = 0.09 > 0.05) group.

### 3.3. mGluR1 and mGluR5 Protein Expression in the Hippocampus

**Figure 4:**
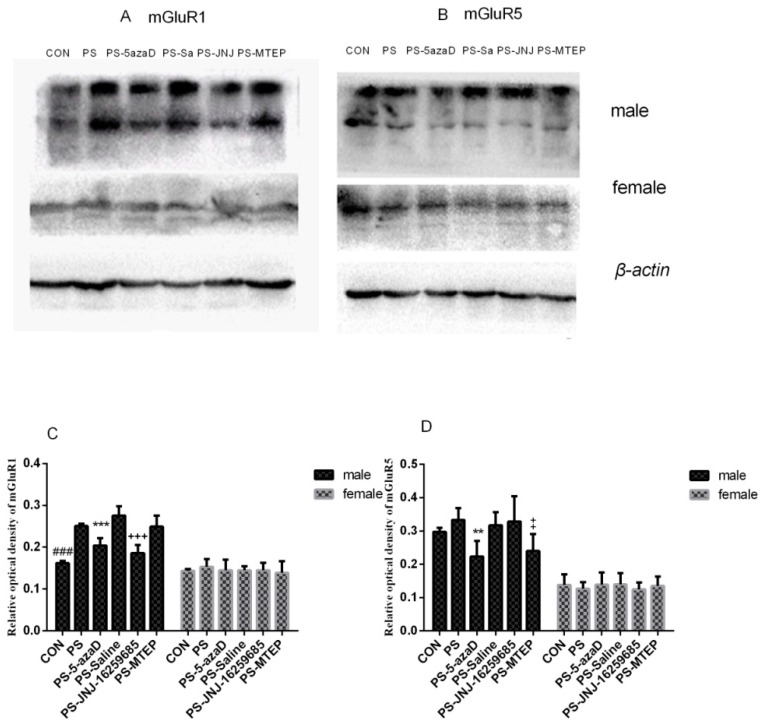
Western Blotting (**A**) MGluR1 changes in OR hippocampus revealed by Western blot; (**B**) MGluR5 changes in OR hippocampus revealed by Western blot; (**C**,**D**) Western blotting analysis of mGluR1 and mGluR5 protein levels in the hippocampus shown in six groups (*n* = 6 each group). Data is expressed as mean ± SEM. ^###^ *p* < 0.001 compare with CON. *** *p* < 0.001, ** *p* < 0.01, ^+++^ *p* < 0.001, 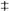 *p* < 0.05 compare with PS.

The effects of PS on the mGluR1 protein expression were investigated. For experiment 3 (Figure 4A), based on the one-way ANOVA, sex differences were observed (F_(1,70)_ = 95.282, *p* < 0.001), as well as the overall effects of male (F_(5,30)_ = 39.636, *p* = 0.027 < 0.05). Our results in the male group analysis showed that, when compared with the CON group, the mGluR1 protein expression in the PS group was remarkably increased (*p* = 0.002 < 0.01), with no difference in the PS-5azaD group (*p* = 0.446 > 0.05). Then, compared with the PS group, the mGluR1 protein expression in the PS-5azaD group (*p* < 0.001) and PS-JNJ group (*p* < 0.001) was significantly decreased, with no difference in the PS-Saline group (*p* = 0.153 > 0.05) and PS-MTEP group (*p* = 0.850 > 0.05). In addition, two-way ANOVA showed that there was no significant difference in mGluR1 protein expression between each female (F_(5,30)_ = 0.378, *p* = 0.860 > 0.05) group.

The effects of PS on the mGluR5 protein expression were investigated. For experiment 3 (Figure 4B), based on the one-way ANOVA, sex differences were observed (F_(1,70)_ = 202.442, *p* < 0.001), as well as the overall effects of male (F_(5,30)_ = 6.493, *p* = 0.045 < 0.05). Our results in the male group analysis showed that, when compared with the CON group, the mGluR5 protein expression was not different in the PS group (*p* = 0.742 > 0.05) and PS-5azaD group (*p* = 0.078 > 0.05). Then, compared with the PS group, the mGluR5 protein expression in the PS-5azaD group (*p* = 0.003 < 0.01) and PS-MTEP group (*p* = 0.014 < 0.05) was significantly decreased, with no difference in the PS-Saline group (*p* = 0.988 > 0.05) and PS-JNJ group (*p* = 0.974 > 0.05). In addition, two-way ANOVA showed that there was no significant difference in mGluR5 protein expression between each female (F_(5,30)_ = 0.312, *p* = 0.902 > 0.05) group.

### 3.4. Promoter Methylation of mGluR1 Gene in the Hippocampus of Offspring

**Figure 5:**
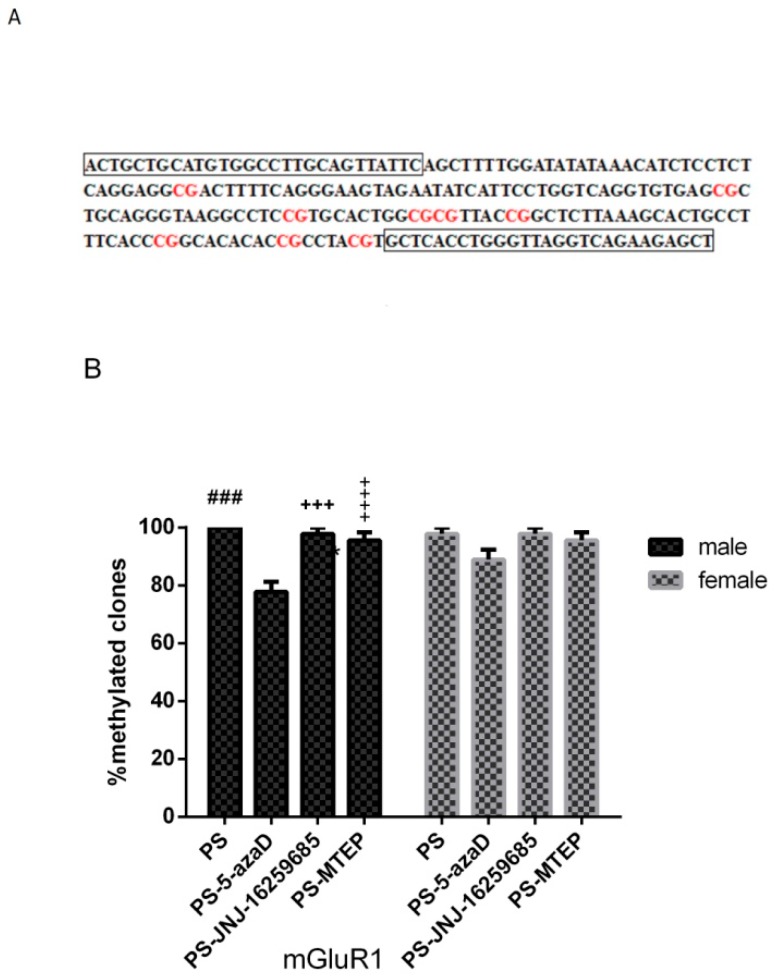
DNA methylation analysis of the mGluR1 gene promoter. (**A**) The design of the primer position is surrounded by the black box; (**B**) Mean + SEM percentage of methylated clones in hippocampal samples from offspring shown in four groups (*n* = 5 each group). ^###^ *p* < 0.001 compare with CON. *** *p* < 0.001, ^+++^ *p* < 0.001, 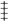 *p* < 0.001 compare with PS.

Based on the above results, the declined mRNA and protein expression levels of mGluR1 and mGluR5 treated by 5-azaD were only observed in the male group. Due to the important role of sex in the pathogenesis of depression and related experiments, the promoter methylation of mGluR1 and mGluR5 gene was subjected to the following investigation, respectively. For experiment 4 (Figure 5B), based on the one-way ANOVA, sex differences were observed (F_(1,38)_ = 0.647, *p* = 0.426 > 0.05), as well as the overall effects of male (F_(3,16)_ = 16.733, *p* < 0.001). Our results in the male group analysis showed that, when compared with the PS group, the promoter methylation of mGluR1 gene in the PS-5azaD group (*p* < 0.001), PS-MTEP group (*p* < 0.001), and PS-JNJ group (*p* < 0.001) was significantly increased. In addition, two-way ANOVA showed that there was no significant difference in promoter methylation of mGluR1 gene between each female (F_(3,16)_ = 2.389, *p* = 0.107 > 0.05) group.

### 3.5. Promoter Methylation of mGluR5 Gene in the Hippocampus of Offspring

**Figure 6:**
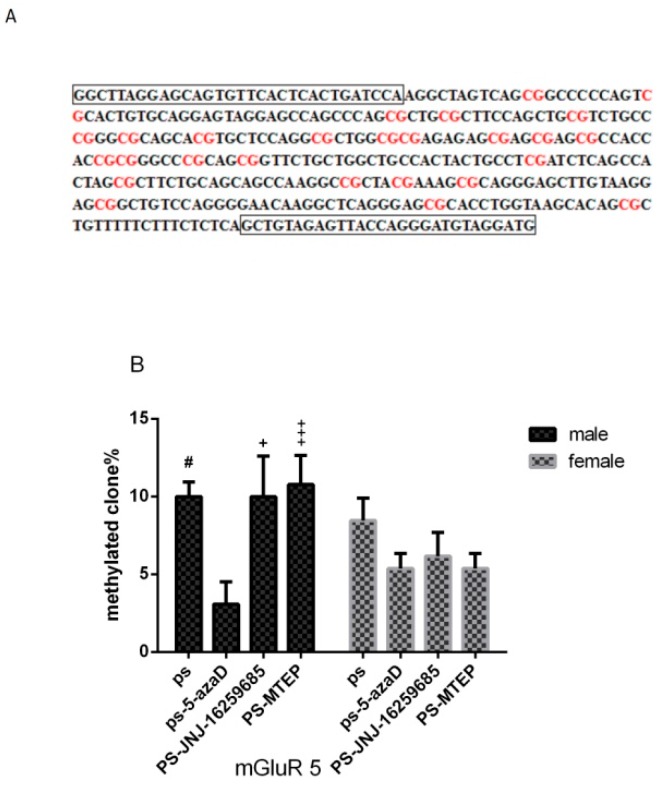
DNA methylation analysis of the mGluR5 gene promoter. (**A**) The design of the primer position is surrounded by the black box; (**B**) Mean + SEM percentage of methylated clones in hippocampal samples from offspring shown in four groups (*n* = 5 each group). ^#^ *p* < 0.05 compare with CON. ^+^ *p* < 0.05, 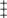 *p* < 0.01 compare with PS.

For experiment 5 (Figure 6B), based on the one-way ANOVA, sex differences were observed (F_(1,38)_ = 2.753, *p* = 0.105 > 0.05), as well as the overall effects of males (F_(3,16)_ = 3.911, *p* = 0.029 < 0.05). Our results in the male group analysis showed that, when compared with the PS group, the promoter methylation of mGluR5 gene in the PS-5azaD group (*p* = 0.016 < 0.05), PS-MTEP group (*p* = 0.009 < 0.01), and PS-JNJ group (*p* = 0.016 < 0.05) was significantly increased. In addition, two-way ANOVA showed that there was no significant difference in promoter methylation of mGluR5 gene between each female (F_(3,16)_ = 1.365, *p* = 0.289 > 0.05) group.

## 4. Discussion

The sucrose preference test (SPT) is one of the most widely used methods for detecting the lack of pleasure in depressive behavior [41]. Anhedonia is an indicator that the sensitivity of the reward response is reduced. In other words, it is considered as the ability to experience pleasure, which is a fundamental feature displayed in clinical depression [42]. The sweet solution is a reward given to the good method. In this study, we discovered that the percentages of sugar water preference in progeny were significantly reduced, and that the administration of 5-azaD was able to effectively interfere with this behavior while also playing a therapeutic role. Sun H and colleagues found that prenatal stress significantly reduced the sugar preference of OR (1%, 32%) [43]. In addition the JNJ and MTEP can significantly increase the sugar preference in both PS offspring male and female rats. The forced swimming test (FST) is a widely used animal model for the detection of depression-like behavior. Moreover, the screening of antidepressant drugs is also an analysis and study of the desperate state of animal behavior. The study conclusively found that prenatal stress can lead to an increase of the immobility of forced swimming in both offspring male and female rats while providing 5azad during pregnancy was able to effectively shorten the time of immobility [44]. These results of this study also reflected that prenatal stress prolongs the immobility of such OR, and that the behavioral administration of the methyltransferase inhibitor 5-azaD prior to the experiment was able to effectively shorten the immobility time. Amanda J Sales et al. also determined that 5-azaD was able to significantly shorten immobility while also producing antidepressant effects [45]. In addition, JNJ can significantly reduce the immobility of forced swimming in both PS offspring male and female rats, and MTEP significantly reduced the immobility of forced swimming only in PS offspring female rats. MGluR1 antagonist EMQMCM could significantly reduce the immobility time in PS OR, but mGluR1 antagonist AIDA had no effect in PS OR [46]. MGluR5 antagonist MPEP and MTEP all showed antidepressant effects in FST and TST mice [47]. There seems to be a gap in the effect of the existing antidepressants, and the potential antidepressant activity of mGluR I antagonist needs further study.

However, in the open field test, our results differed from previous reports and findings. Although the central residence time of the offspring after prenatal stress was greatly reduced in the central residence time, the central residence time increased within the 5-azaD treatment group. Moreover, we were unable to observe any considerable differences in rearing as well as in the frequency of micturition. Our open field test protocol and those previously reported should be identical, so this inconsistency may be a result of experimental settings with subtly varying conditions, for instance, rat varieties, stress models, experimental methods, square size, box height, or perhaps even the differences in analysis methods. In summary, we conclude that 5-azaD was able to effectively intervene in prenatal stress leading to offspring depression and anxiety like behaviors. This supports the fact that it plays a consequential role of the depressive behavior against 5-azaD. Our experimental results have determined that there was a gender difference in the expression of mGluR1 and mGluR5 within the hippocampus. Prenatal stress increased the mRNA and protein expression of mGluR1 and mGluR5 in the hippocampus of depressed male offspring, which were downregulated by 5-azaD. Epigenetic modifications induced by PS might result in a series of adaptive mechanisms that are not apparently consistent with behavioral changes [23].

A large amount of evidence has shown that glutamate and glutamate receptors play a vital role in the pathogenesis and antidepressant treatment of depression in the central nervous system [8]. Previous studies showed that prenatal stress significantly increased the expression of mGluR2/3 and mGluR5 in the prefrontal cortex of male OR [48]. Three subunits of the G protein coupled with the glutamate receptors (metabotropic glutamate receptor mGluRs I, II, and III) [10] were confirmed to be associated with stress responses. Moreover, a large number of experimental data indicated that stress response leads to an increase in metabolic glutamate receptor I (mGluR1 and mGluR5) as well as in protein levels within the hippocampus [49,50]. The mGluR2 and mGluR3 can reduce the transfer of excitatory glutamic acid by inhibiting its own receptor. The mGluRl and mGluR5 are located in the terminal of the inhibitory intermediate neurons. The mGluRl and mGluR5 upregulation enhances excitatory glutamate transmission and cell excitability [51]. Our findings showed that there were significant changes in the mRNA or protein expression levels of mGluR1 and mGluR5 in the hippocampus in the PS male OR, while not the female OR. The microinjection of 5-azaD significantly reversed the declined expression levels of mGluRl and mGluR5 in the hippocampus in the PS male OR, while not the female OR. Many other studies have shown that the mGluR5 receptor antagonist (MTEP) has a significant antidepressant effect [52], and the mGluR1 receptor antagonist (JNJ-16259685) has a significant antidepressant effect which also supports our findings [53]. The expression of mGluR5 significantly increased in the hippocampus of experimental rats after taking agomelatine [54]. This is considered a compensation mechanism for mGluRl and mGluR5 insensitive to this antidepressant [55].

Recent studies have found that epigenetic mechanisms of methylation modification are involved in the pathogenesis of depressive-like behavior [56]. More specifically, the methylation in the gene regulatory region was to silence the expression of the gene [57]. High methylation modification leads to high methylation level, whereas 5-azaD could decrease the methylation level and ameliorate depressive behavior [45]. Our results showed that the mGluR1 and mGluR5 gene promoter methylation level was significantly elevated in the hippocampus of the PS OR, indicating that the methylation might be related to the pathogenesis of depression-like behavior. Moreover, our results showed that the methylation level of mGluR1 and mGluR5 gene promoter was significantly reduced in the hippocampus in the PS-5azaD group in male OR, while not the female OR. More interestingly, after the PS exposure, the mGluR1 and mGluR5 gene promoter methylation level in the hippocampus in the OR was not significantly changed when treat with JNJ and MTEP. These phenomena might be explained that JNJ and MTEP participate in the improvement of depressive behavior by inhibition of the expression of mGluR1 and mGluR5 while not the reduction of promoter methylation. High promoter methylation modification level blocked the incorporation of the relevant transcriptional elements to the mGluR1 and mGluR5 promoter region, thus inhibiting the transcription of the mGluR1 and mGluR5 mRNA and leading to depression-like behavior in the male OR, which could be reversed by the microinjection of 5-azaD.

Gender differences might be explained by the fact that PS influences the epigenetic regulation in a sex specific manner in the hippocampus of the male and female OR [58]. Moreover, the expression of DNMT1 in normal male placenta was lower than that of female. Early stress increased the expression of DNMT1 in female placenta, rather than in the male placenta [59]. This suggests that gender-specific regulation of maternal placenta may be affected by epigenetic modification, and that the growth factors associated with placental gene expression, nutritional transport, and epigenetic changes may both affect the development of the central nervous system. Previous studies have shown that the maintenance of methylation patterns is critical in terms of neurodevelopment. Placental DNA methylation disorders are a predictor of fetal neurodevelopment. Furthermore, stress-induced increase in DNMT1 expression suggests that women can maintain methylation by maintaining methylation patterns to withstand the effects of stress [60,61].

Further in depth issues should be addressed in the future. The epigenetic regulation of depression-related genes is very important for the PS induced depression-like behavior in offspring [62]. Therefore, the relationship between the mGluR1 and mGluR5 gene promoter methylation modification of housekeeping genes and depression-like behavior in the OR needs to be discussed in details.

In conclusion, our results suggest that the mGluR1 and mGluR5 DNA methylation modification in the male OR hippocampus after PS might be involved in the epigenetic mechanism for the pathogenesis of depression-like behavior, in a sex specific manner. Our findings provide evidence for the epigenetic and sex-specific contribution of mGluR1 and mGluR5 gene promoter methylation to the development of PS induced depression-like behavior in the male OR, as well as the disease treatment.

## Figures and Tables

**Table 1:** Primer sequences and PCR conditions. All templates were initially denatured for 3 min at 94 °C, and after completing all cycles, were extended a final extension of 5 min at 72 °C.

mRNA	Primer(5′-3′)	Denatring °C (s)	Annealing °C (s)	Elongation °C (s)	Cycles
mGluR1		94 (30)	56 (30)	72 (60)	39
F:GGTCCCTTCTGACACTTTGC					
R:AAGCATCCATTCCACTCTCG					
mGluR5		94 (30)	56 (30)	72 (60)	39
F:TGAGAGGAAGTGTGGTGCAG					
R:CAAGTGTGATGTTGGGCAAG					

**Table 2:** Primer sequences and PRC conditions. All templates were initially denatured for 4 min at 98 °C, and after completing all cycles, were extended a final extension of 8 min at 72 °C.

mRNA	Primer (5′-3′)	Denatring °C (s)	Annealing °C (s)	Elongation °C (s)	Cycles
mGluR1		94 (45)	66 (45)	72 (60)	20
F:ATTGTTGTATGTGGTTTTGTAGTTATTT					
R:AACTCTTCTAACCTAACCCAAATAAAC					
mGluR5		94 (45)	56 (45)	72 (60)	20
F:GGTTTAGGAGTAGTGTTTATTTATTGATTTA					
R:CATCCTACATCCCTAATAACTCTACAAC					
